# Extract From *Plectranthus amboinicus* Inhibit Maturation and Release of Interleukin 1β Through Inhibition of NF-κB Nuclear Translocation and NLRP3 Inflammasome Activation

**DOI:** 10.3389/fphar.2019.00573

**Published:** 2019-05-28

**Authors:** Wohn-Jenn Leu, Jui-Ching Chen, Jih-Hwa Guh

**Affiliations:** ^1^School of Pharmacy, National Taiwan University, Taipei, Taiwan; ^2^Oneness Biotech Co., Ltd., Taipei, Taiwan

**Keywords:** *Plectranthus amboinicus*, rosmarinic acid, cirsimaritin, carvacrol, NLRP3 inflammasome

## Abstract

Uncontrolled inflammation may produce massive inflammatory cytokines, in which interleukin 1β (IL-1β) plays a key role, resulting in tissue damage and serious disorders. The activation of NLRP3 inflammasome is one of the major mechanisms in maturation and release of IL-1β. *Plectranthus amboinicus* is a perennial herb. Several pharmacological activities of natural components and crude extracts from *P. amboinicus* have been reported including anti-inflammation; however, the underlying mechanism is not clear. Phorbol-12-myristate 13-acetate-differentiated THP-1 monocytic leukemia cells were used as a reliable model in this study to examine the effect on inflammasome signaling pathway by PA-F4, an extract from *Plectranthus amboinicus*. PA-F4 inhibited ATP-induced release of caspase-1, IL-1β, and IL-18 from lipopolysaccharides (LPS)-primed cells. PA-F4 induced a concentration-dependent inhibition of both ASC dimerization and oligomerization in cells under LPS priming plus ATP stimulation. Co-immunoprecipitation of NLRP3 and ASC demonstrated that PA-F4 significantly blunted the interaction between NLRP3 and ASC. Furthermore, PA-F4 completely abolished ATP-induced K^+^ efflux reaction in LPS-primed cells. Taken together, PA-F4 displayed an inhibitory activity on NLRP3 inflammasome activation. Moreover, PA-F4 also inhibited LPS-induced p65 NF-κB activation, suggesting an inhibitory activity on LPS priming step. Further identification showed that rosmarinic acid, cirsimaritin, salvigenin, and carvacrol, four constituents in PA-F4, inhibited LPS-induced IL-6 release. In contrast, rosmarinic acid, cirsimaritin and carvacrol but not salvigenin inhibited ATP-induced caspase-1 release from LPS-primed cells. In conclusion, PA-F4 displayed an inhibitory activity on activation of NLRP3 inflammasome. PA-F4 inhibited LPS priming step through block of p65 NF-κB activation. It also inhibited ATP-induced signaling pathways in LPS-primed cells including the inhibition of both ASC dimerization and oligomerization, K^+^ efflux reaction, and the release reaction of caspase-1, IL-1β, and IL-18. Rosmarinic acid, cirsimaritin, salvigenin, and carvacrol could partly explain PA-F4-mediated inhibitory activity on blocking the activation of NLRP3 inflammasome.

## Introduction

Inflammation is a double-edged sword. It plays a key role on triggering host defense response through removing pathogens. However, uncontrolled inflammation can produce massive inflammatory cytokines which may cause tissue damage, leading to serious disorders (Pedraza-Alva et al., 2015; Vince and Silke, 2016). Interleukin (IL) 1β is a key inflammatory cytokine participating in the generation of systemic and local responses to infection and injury. Overproduction of IL-1β may also cause a variety of different autoinflammatory syndromes. IL-1β maturation needs an intracellular multimeric protein complex called inflammasome in which NLRP3 inflammasome is the most widely studied inflammasome that consists of the NLRP3 protein, the ASC adaptor and procaspase-1 (Elliott and Sutterwala, 2015). Upon stimulation, the inflammasome components assemble into a large complex where NLRP3 and ASC adhere through the PYD domains, whereas ASC and procaspase-1 adhere through the CARD domains (Vajjhala et al., 2012; Proell et al., 2013). The caspase-1, which is activated after autocleavage in the NLRP3 inflammasome, can eventually lead to the maturation and secretion of IL-1β (Vajjhala et al., 2012; Proell et al., 2013; Elliott and Sutterwala, 2015; Tőzsér and Benkő, 2016).

NF-κB family consists of several inducible transcription factors which control immune and inflammatory effects in response to a variety of cellular stresses. Nuclear translocation and activation of NF-κB contribute to a massive increase of expression of inflammatory cytokines such as TNF-α, IL-1β and IL-6. Furthermore, NF-κB is also responsible for the activation of NLRP3 expression since this activation can be inhibited by NF-κB activation inhibitors or by infection with an adenoviral expression vector constitutively expressing a superrepressor of NF-κB (Boaru et al., 2015). The studies suggest that NF-κB is a key mediator on the priming effect of NLRP3 inflammasome activation.

*Plectranthus amboinicus* is a perennial herb occurring naturally throughout the tropics and warm regions of Africa, Asia, and Australia (Arumugam et al., 2016). Several pharmacological activities of natural components and crude extracts from *P. amboinicus* have been reported including anti-inflammatory, anti-bacterial, anti-epileptic, larvicidal and analgesic activities (Senthilkumar and Venkatesalu, 2010; Chen et al., 2014; Arumugam et al., 2016). Furthermore, several studies have addressed the wound healing activity of *P. amboinicus* in *in vivo* models through immune-stimulation, enhancing epithelialization and collagen deposition, and increasing wound contraction (Jain et al., 2012; Arumugam et al., 2016). However, the underlying molecular mechanism of *P. amboinicus* on the inhibition of inflammation is unclear. In this study, the plants of *P. amboinicus* were collected according to good agricultural and good collection practices. Guided by previous pharmacological studies (Gurgel et al., 2009; Chiu et al., 2012; Kuo et al., 2012), the most active fractions, PA-F4 from *P. amboinicus* were obtained (Kuo et al., 2012). This study has been conducted to shed light on the mechanism of PA-F4 in both NF-κB and NLRP3 inflammasome involved signaling pathway.

## Materials and Methods

### Materials

RPMI 1640 medium, PSA Solution (100 U/ml penicillin and 0.1 mM Streptomycin and 250 nM Amphotericin B) and fetal bovine serum (FBS), sodium pyruvate were obtained from GIBCO/BRL Life Technologies (Grand Island, NY, United States). Antibodies to α-tubulin, NF-κB p65, and HRP-conjugated anti-mouse and anti-rabbit IgG were obtained from Santa Cruz Biotechnology, Inc. (Santa Cruz, CA, United States). Antibodies to Caspase-1, ASC, NLRP3, p-p44/42 MAPK (Erk1/2)^Thr202/Tyr204^, p44/42 MAPK (Erk1/2), p-p38 MAPK^Thr180/Tyr182^, p-IκB^Ser32^, IκB, p-c-Jun^Ser63^, and GAPDH were from Cell Signaling Technologies (Boston, MA, United States). Antibodies to p-JNK1/2/3^(Y 185/Y 185/Y 223)^, were from ABCam (Cambridge, MA, United States). Lipopolysaccharides (LPS), adenosine triphosphate (ATP), trichloroacetic acid (TCA), acetone, D-glucose, NaHCO_3_, dithiothreitol, phenylmethylsulfonylfluoride (PMSF), MTT, leupeptin, NaF, NaVO_4_, disuccinimidyl suberate (DSS), CHAPS, and all other chemical compounds were obtained from Sigma-Aldrich (St. Louis, MO, United States). Human Caspase-1/ICE (DCA100) and IL-1β/IL-1F2 (DLB50) Immunoassay Kits were from R&D system (Minneapolis, MN, United States). Human IL-18 ELISA kit was from MBL (Nagoya, Japan). Bio-Red protein assay kit was from Bio-Red (Hercules, CA, United States). PA-F4 extracted from *P. amboinicus* and its constituents including rosmarinic acid (purity of 100%), cirsimaritin (purity of 96.1%), salvigenin (purity of >98%) and carvacrol (purity of 99.9%) were purchased from USP (Rockville, MD, United States), Green Chem (Nantou, Taiwan), Green Chem (Nantou, Taiwan) and Tokyo Chemical Industry (Tokyo, Japan), respectively.

### Cell Lines and Cell Culture

THP-1, a human monocytic cell line derived from acute monocytic leukemia, was obtained from the Bioresources Collection and Research Center (BCRC) of the Food Industry Research and Development Institute (Hsinchu, Taiwan). Cells were cultured in RPMI 1640 medium containing 10% inactivated fetal bovine serum, 100 U/ml penicillin and 0.1 mM Streptomycin and 250 nM Amphotericin B and 2.5 g/L glucose and 1 mM sodium pyruvate. Cells were maintained in a humidified incubator at 37°C in 5% CO_2_/95% air.

### MTT Assays

Cells were seeded in 96-well plates and differentiated by 50 nM PMA to macrophage. After 48 h, cells were co-treated with 1 μg/mL LPS and indicated agent for 1 h, and then added 0.5 mg/mL MTT (dissolved in PBS) for 2 h. After the treatment, the formed formazan was dissolved in 0.1 mL DMSO for 5 min. The absorbance was read at a wavelength of 590 nm.

### Cytokine Release

Cells were seeded and differentiated by 50 nM PMA in 48-well plates. After 48 h, cells were primed with 1 μg/mL LPS for 4 h. Then, cells were pre-treated with the indicated agent for 0.5 h and then co-treated with 5 mM ATP and the indicated agent for another 2 h. After treatment, cytokine levels in the medium were quantified using quantikine ELISA kits according to manufacturer’s protocols. Briefly, test medium was added to the wells of microplate which was precoated with a monoclonal antibody specific for the target cytokine. After a 2-h incubation at room temperature and washing, specific cytokine conjugate was added for further 1-h incubation. After washing, substrate solution was added to each well for another 20-min incubation at room temperature. Finally, stop solution was added to each well. The color was developed and the optical density was determined.

### Western Blotting

After the treatment, the cells or the medium were obtained. For cellular protein, the cells were lysed in 0.1 ml of lysis buffer (10 mM Tris-HCl pH 7.4, 150 mM NaCl, 1 mM EGTA, 1% Triton X-100, 1 mM phenylmethylsulfonyl fluoride, 10 μg/ml leupeptin, 1 mM DTT, 50 mM sodium fluoride and 1 mM sodium orthovanadate) for 30 min at 4°C. After centrifugation, the supernatants were collected and the concentration of protein was quantified. For protein in the medium was precipitated by 20% TCA for 30 min on ice. After centrifugation for 20 min at 12,000 rpm, the pellets were washed twice by acetone. For Western blot analysis, 30 μg proteins were separated by electrophoresis in a 10% or 14% polyacrylamide gel and transferred to a PVDF membrane. After 1 h incubation at room temperature in PBS/5% non-fat milk, the membrane was washed with PBS/0.1% Tween 20 for another 1 h and overnight incubated with the indicated antibody at 4°C. After three washings with PBS/0.1% Tween 20, the anti-mouse or anti-rabbit IgG (dilute 1:8000) was applied to the membranes for 1 h at room temperature. The membranes were washed with PBS/0.1% Tween 20 for 1 h. The detection of signal was performed with an enhanced chemiluminescence detection kit (Amersham, Buckinghamshire, United Kingdom) and the membranes were scanned using a ChemiDoc^TM^ MP Imaging System.

### ASC Oligomerization

After the treatment, cells were re-suspended in 0.1 ml buffer A (20 mM HEPES pH 7.4, 10 mM KCl, 1.5 mM MgCl_2_, 1 mM EGTA, 1 mM EDTA, 1 mM phenylmethylsulfonyl fluoride, 10 μg/ml leupeptin, 1 mM sodium fluoride, and 1 mM sodium orthovanadate) and lysed by shearing 30 times through a 27-gauge needle. 0.02 mL of lysate was collected acting as loading input protein. Lysates were centrifuged at 300 ×*g* for 8 min at 4°C. Supernatants were collected and diluted with equal volume of CHAPS lysis buffer and then centrifuged at 2,600 ×*g* for 8 min at 4°C. Supernatants were discarded and re-suspended the pellets in 0.02 mL of lysis buffer containing 4 mM of DSS. Samples were incubated for 30 min at room temperature to cross-link proteins. Samples were mixed with 5× sample buffer and boiled at 90°C for 5 min.

### Measurement of Intracellular Potassium Content

After treatment, cells were re-suspended in 0.3 mL ddH_2_O and sonicated in ice water bath (power: 300 W for 3 s, interval for 30 s, repeat for 5 times). The prepared homogenate liquid kept for detection without centrifugation. After sonication, the protein concentration of homogenate liquid was quantified and the intracellular potassium content was quantified using Potassium Assay Kit (MyBioSource, San Diego, CA, United States) according to manufacturer’s protocols. Briefly, the commercial protein precipitant was added into 20 μL of homogenate liquid. After centrifugation, the supernatant was obtained and was mixed with working solution for 5 min. The optical density of each well was determined at 450 nm. Data were normalized based on the protein concentration of the homogenate liquid.

### Immunoprecipitation Assay

After the treatment, the cells were trypsinized and centrifuged at 2,000 rpm for 10 min at 4°C. The cell pellet was resuspended in 0.5 ml IP lysis buffer (50 mM HEPES, pH 7.4, 150 mM NaCl, 10% Glycerol, 2 mM EDTA, 0.5% Triton X-100, 1 mM phenylmethylsulfonyl fluoride, 10 μg/ml leupeptin, 1 mM DTT, 50 mM sodium fluoride and 1 mM sodium orthovanadate) for 30 min on ice and then centrifuged at 12,000 rpm for 20 min at 4°C. The protein concentration of supernatant was quantified. The 500 μg supernatant was immunoprecipitated with the 1 μg antibody against ASC at 4°C overnight. A/G magnetic beads were added to each sample at 4°C overnight. The beads were washed four times with IP lysis buffer. Beads-bound proteins were mixed with 2× sample buffer and boiled at 90°C for 5 min for immunoblotting.

### Data Analysis

Data are presented as the mean ± SEM for the indicated number of separate experiments. One-way ANOVA followed by a Newman–Keuls *post hoc* test is applied. *P*-values less than 0.05 are statistically considered significant.

## Results

### PA-F4 Inhibits the Release of Processed Caspase-1 p20 Subunit, IL-1β and IL-18

**FIGURE 1:**
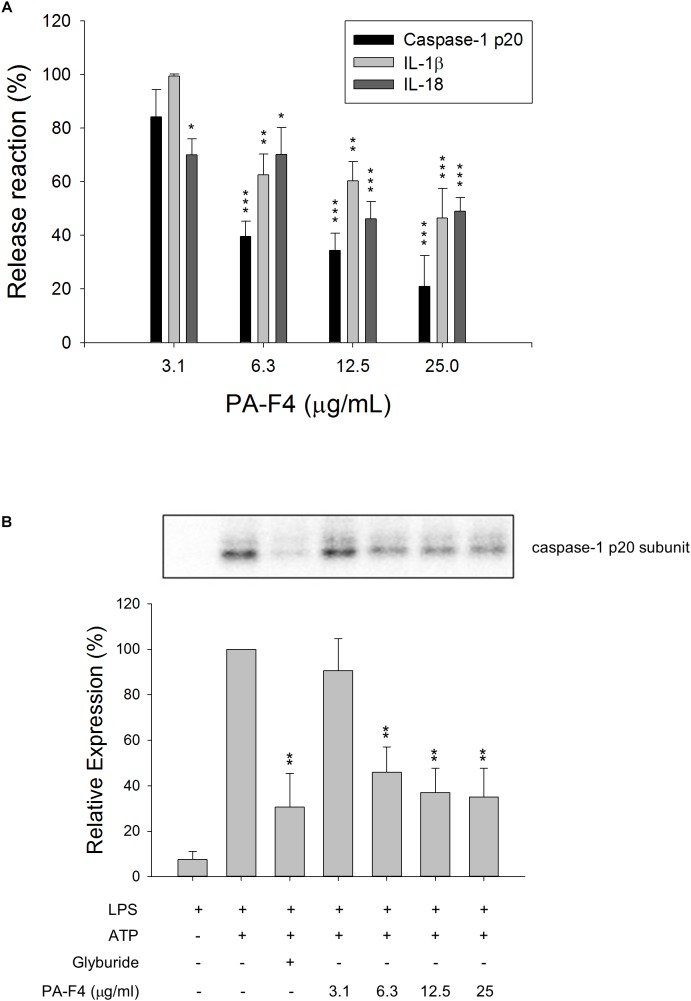
Effect of PA-F4 on the release reaction of caspase-1 and cytokines and caspase-1 expression. THP-1 cells were seeded and differentiated by 50 nM PMA. After 48 h, cells were primed with 1 μg/mL LPS for 4 h. Then, cells were pre-treated with or without the indicated agent for 0.5 h and then co-treated with 5 mM ATP for another 2 h. After treatment, the levels of p20 caspase-1 subunit and cytokines in the medium were quantified **(A)** or the p20 caspase-1 subunit expression was detected **(B)**. Data are expressed as mean ± SEM of three independent experiments. One-way ANOVA by Newman–Keuls *post hoc* test is used. ^∗^*P* < 0.05, ^∗∗^*P* < 0.01, ^∗∗∗^*P* < 0.001 compared with control medium **(A)** or with LPS plus ATP **(B)**. The analysis of *P* (ANOVA) indicates *P* < 0.001 in both **(A,B)**.

Lipopolysaccharides priming followed by subsequent exogenous ATP-induced activation of P2X_7_ receptor represents a typical model for the activation of NLRP3 inflammasome and is well characterized to induce autoproteolysis of pro-protein caspase-1 into activated caspase-1, which leads to the cleavage of pro-IL-1β and pro-IL-18 into their active and secreted forms (Kahlenberg et al., 2005; Ghonime et al., 2014). THP-1 monocytic leukemia cells which were differentiated into macrophages using PMA were used as a reliable model to study the regulation of inflammasome signaling pathway. To understand the effect of PF-F4 on inflammasome activation and related downstream signaling, the cells were primed with LPS for 4 h and then, the cells were treated with PA-F4 in the absence or presence of ATP. The data demonstrated that ATP addition induced the release of processed caspase-1 p20 subunit into the culture medium. This effect was inhibited by PA-F4 in a concentration-dependent fashion determined using an ELISA system (Figure 1A). Western blotting analysis further demonstrated a more than 10-fold increase in LPS priming while subsequently applied ATP in differentiated THP-1 cells. Both glibenclamide (a positive control) and PA-F4 significantly inhibited the release reaction of caspase-1 p20 subunit (Figure 1B). Furthermore, the release of IL-1β and IL-18 determined using an ELISA system also was inhibited by PA-F4 (Figure 1A). Western blotting analysis further showed that PA-F4 inhibited the formation of active form of IL-1β (Supplementary Figure S1). The results indicate that PA-F4 may display an inhibitory activity on inflammasome activation.

### PA-F4 Displays Inhibitory Activity on LPS Priming Step

**FIGURE 2:**
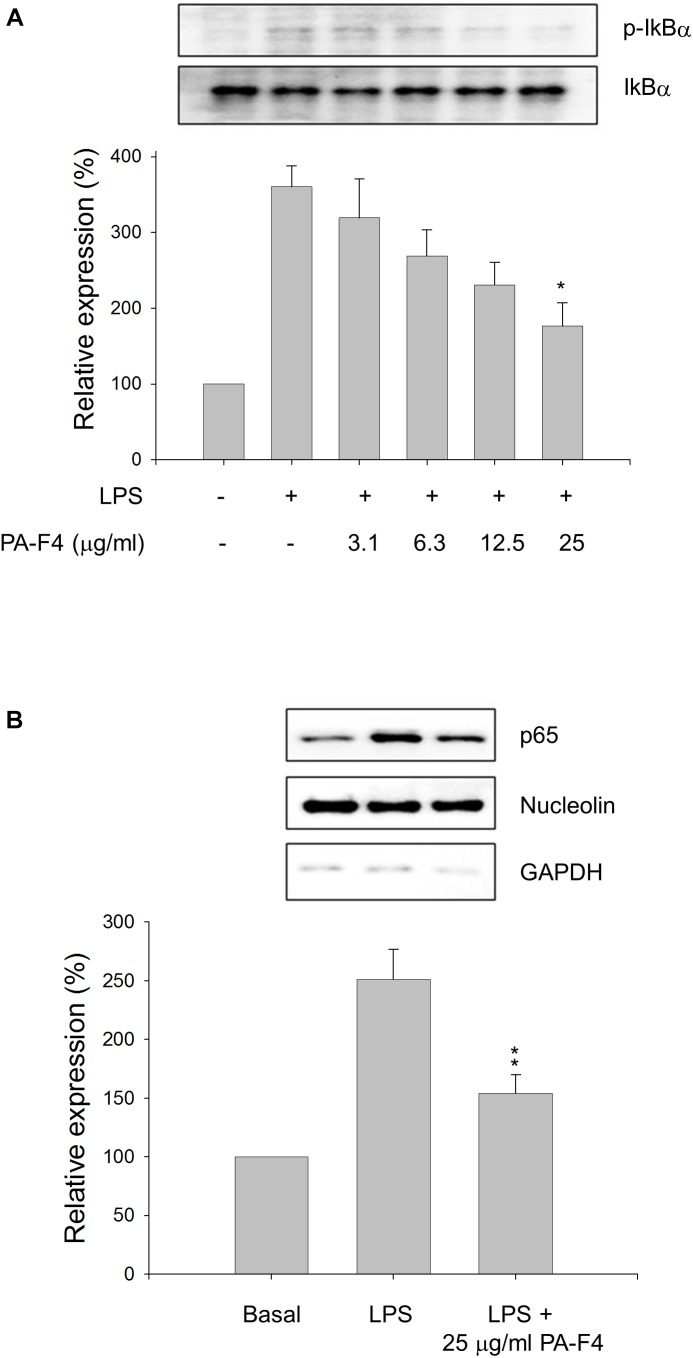
Effect of PA-F4 on LPS-induced IκBα phosphorylation and p65 NF-κB nuclear translocation. THP-1 cells were seeded and differentiated by 50 nM PMA. After 48 h, the cells were pre-treated in the absence or presence of the indicated agent for 0.5 h and then co-treated with 1 μg/ml LPS for 2 h **(A)** or 0.5 h **(B)**. The cells were harvested for the detection of p-IkBα and IkBα **(A)** or nuclear translocation of p65 NF-κB **(B)** using Western blot analysis. Data are expressed as mean ± SEM of three independent determinations. One-way ANOVA by Newman–Keuls *post hoc* test is used. ^∗^*P* < 0.05, ^∗∗^*P* < 0.01 compared with LPS control. The analysis of *P* (ANOVA) indicates the following: for p-IkB group, *P* = 0.001; for p65 NF-κB group, *P* = 0.002.

Inflammasome activation occurs in two steps. First, the Toll-like receptors (TLRs) signaling or inflammatory cytokines can induce nuclear factor κB (NF-κB)-dependent gene expression encoding NLRP3, pro-IL-1β, and pro-IL-18. The second signal is the stimulation of inflammasome assembly, leading to caspase-1 activation and processing of peo-IL-1β and pro-IL-18 into active forms. In the first step, the phosphorylation and degradation of IκBα and subsequent nuclear translocation of NF-κB transcription factor are key events in response to LPS stimulation. The data in Figure 2A showed that LPS induced a dramatic increase of IκBα phosphorylation in differentiated THP-1 cells and the effect was inhibited by PA-F4 in a concentration-dependent fashion. Furthermore, LPS induced a profound increase of nuclear translocation of p65 NF-κB which was significantly inhibited in the presence of PA-F4 (Figure 2B). The data suggest that PA-F4 displays an inhibitory activity on the LPS priming step.

### PA-F4 Shows Different Regulation on LPS-Primed MAPK Pathways

**FIGURE 3:**
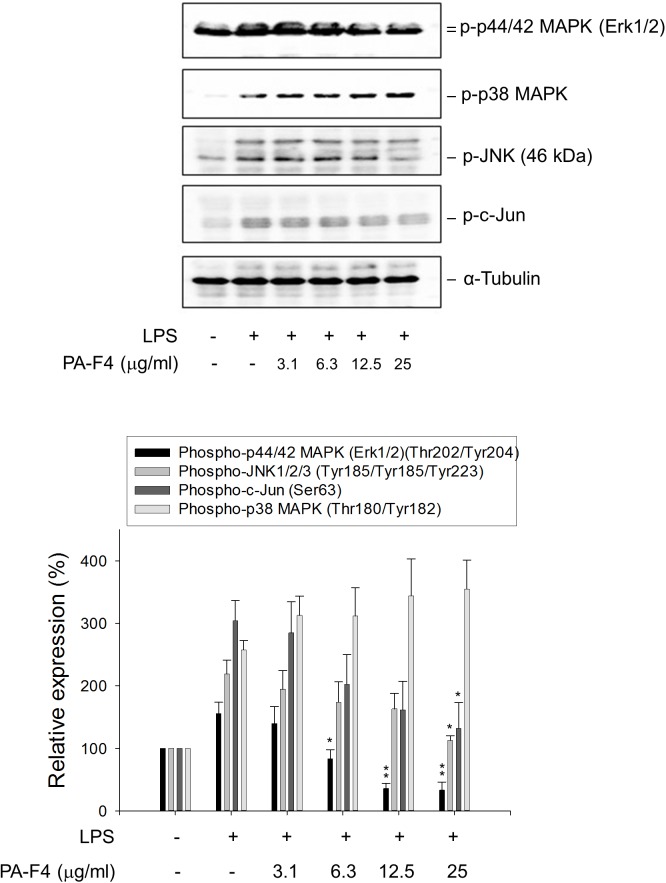
Effect of PA-F4 on LPS-induced MAPK phosphorylation. THP-1 cells were seeded and differentiated by 50 nM PMA. After 48 h, the cells were pre-treated in the absence or presence of the indicated agent for 0.5 h and then co-treated with 1 μg/ml LPS for 2 h. The cells were harvested for the detection of the indicated protein expression using Western blot analysis. Data are expressed as mean ± SEM of three to five independent determinations. One-way ANOVA by Newman–Keuls *post hoc* test is used. ^∗^*P* < 0.05, ^∗∗^*P* < 0.01 compared with LPS control. The analysis of *P* (ANOVA) indicates the following: for phospho-p44/42 MAPK group, *P* < 0.001; for phospho-JNK1/2/3 group, *P* = 0.008; for phospho-c-Jun group, *P* = 0.010; for phospho-p38 MAPK group, *P* = 0.005.

Activation of MAPKs by LPS has been extensively studied to involve TLRs and seems to be differentially regulated in different cell lines and in different expression of receptors and signal transduction molecules. It has been suggested that NLRP3 inflammasome priming requires the activation of MAPKs, in particular the ERK activation (Ghonime et al., 2014). The data in Figure 3 demonstrated that LPS priming induced the activation of c-Jun and MAPKs including ERK, c-Jun N-terminal kinase (JNK) and p38 MAPK in differentiated THP-1 cells. Notably, PA-F4 displayed different regulation in these MAPK pathways, showing inhibitory effect on both ERK and JNK pathways but stimulatory effect on p38 MAPK (Figure 3).

### PA-F4 Inhibits the ASC-Dependent Inflammasome Formation

**FIGURE 4:**
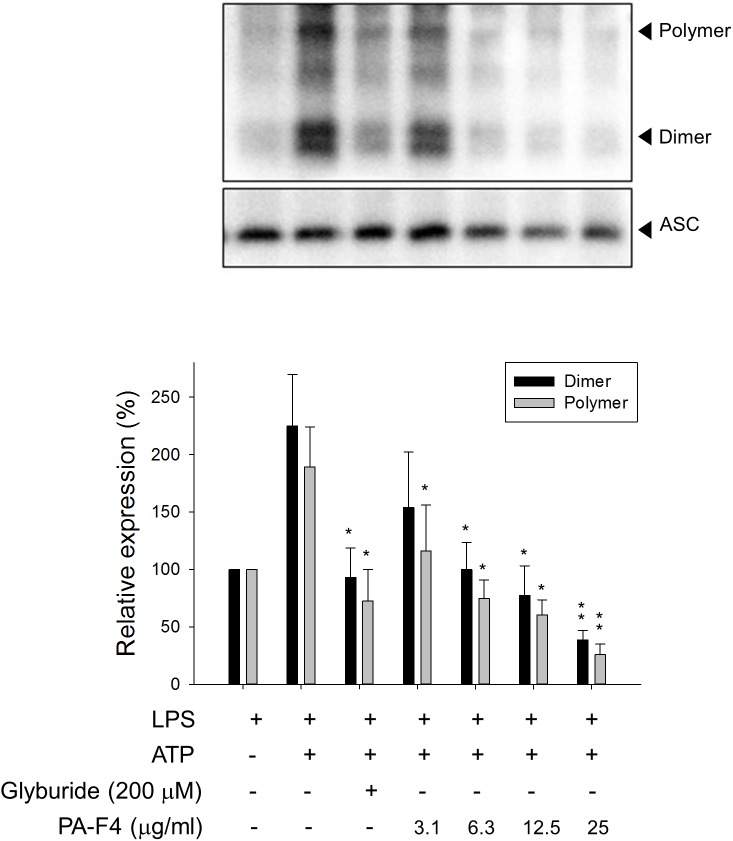
Effect of PA-F4 on ATP-induced ASC dimerization and oligomerization. THP-1 cells were seeded and differentiated by 50 nM PMA. After 48 h, cells were primed with 1 μg/mL LPS for 4 h. Then, cells were pre-treated with or without the indicated agent for 0.5 h and then co-treated with 5 mM ATP for another 2 h. After treatment, the cells were harvested for the detection of the protein expressions of ASC dimerization and oligomerization. Data are expressed as mean ± SEM of four to five independent determinations. One-way ANOVA by Newman–Keuls *post hoc* test is used. ^∗^*P* < 0.05, ^∗∗^*P* < 0.01 compared with LPS plus ATP. The analysis of *P* (ANOVA) indicates the following: for dimer group, *P* < 0.001; for polymer group, *P* = 0.004.

**FIGURE 5:**
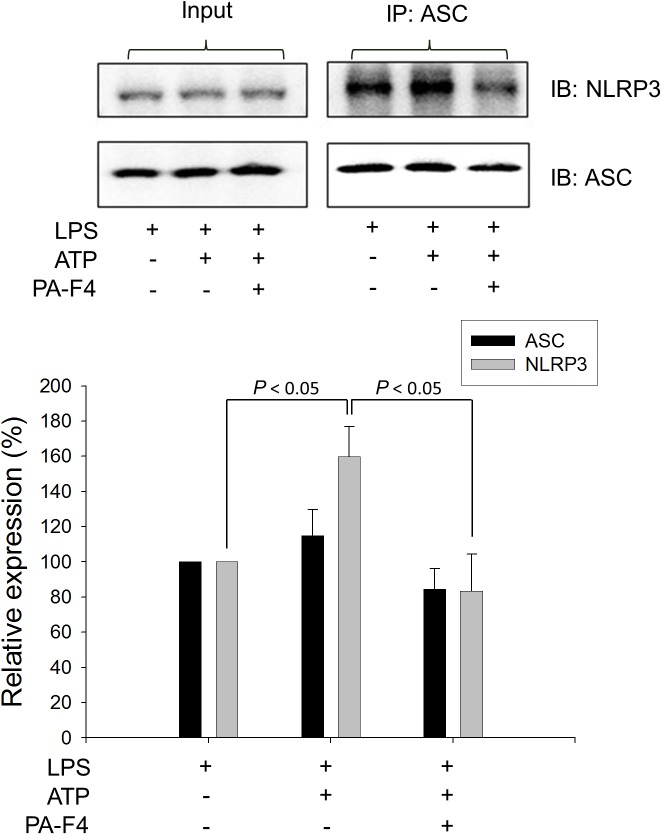
Effect of PA-F4 on ATP-induced interaction between NLRP3 and ASC. THP-1 cells were seeded and differentiated by 50 nM PMA. After 48 h, cells were primed with 1 μg/mL LPS for 4 h. Then, cells were pre-treated with or without PA-F4 (25 μg/ml) for 0.5 h and then co-treated with 5 mM ATP for another 2 h. After the treatment, immunoprecipitation and immunoblotting experiments were performed. Data are expressed as mean ± SEM of three independent determinations. One-way ANOVA by Newman–Keuls *post hoc* test is used. The analysis of *P* (ANOVA) indicates *P* = 0.016.

Upon inflammasome activation, NLRP3 associates ASC adaptor protein which has a dual PYRIN-CARD (caspase recruitment domain) structure allowing the recruitment of pro-caspase-1 to form active inflammasome complexes (NLRP3/ASC/pro-caspase-1) (Srinivasula et al., 2002; Sharma and Kanneganti, 2016). Both ASC dimerization and oligomerization are thought to be direct evidence for inflammasome activation (Lugrin and Martinon, 2017). PA-F4 showed a concentration-dependent inhibition of both ASC dimerization and oligomerization in differentiated THP-1 cells under LPS priming and ATP applied stimulation. Similar effect was also observed in the presence of glibenclamide (a positive control) (Figure 4). Furthermore, the activation of NLRP3 is suggested to create the molecular base that assembles the formation of helical fibrils of ASC via pyrin domain interactions (Lu et al., 2014). We co-immunoprecipitated NLRP3 and ASC and found a profound increase of the interaction during inflammasome activation. PA-F4 significantly blunted the interaction between NLRP3 and ASC (Figure 5), indicating the inhibition of the assembly mechanism.

### PA-F4 Inhibits ATP Induced K^+^ Efflux in LPS-Primed Macrophages

**FIGURE 6:**
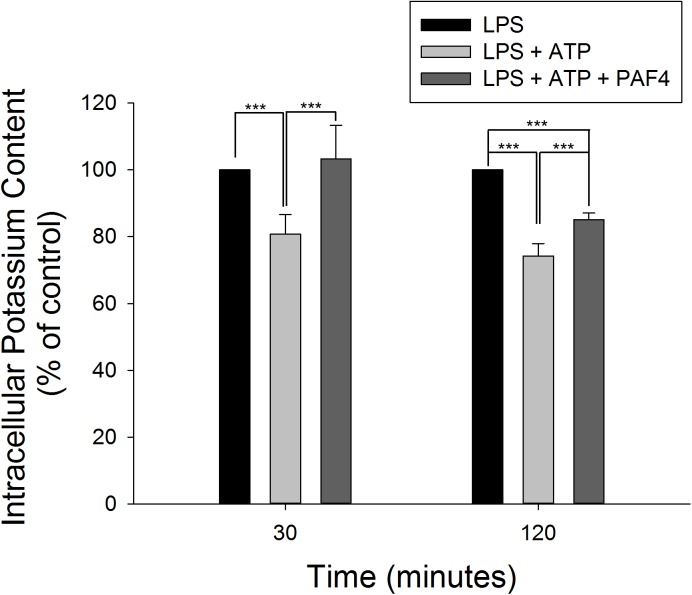
Effect of PA-F4 on ATP-induced K^+^ outflow from cells. THP-1 cells were seeded and differentiated by 50 nM PMA. After 48 h, cells were primed with 1 μg/mL LPS for 4 h. Then, cells were pre-treated with or without PA-F4 (25 μg/ml) for 0.5 h and then co-treated with 5 mM ATP for another 30 min or 2 h. After treatment, intracellular potassium content was detected using Potassium Assay Kit (MyBioSource, San Diego, CA, United States) according to manufacturer’s protocols. Data are expressed as mean ± SEM of four to five independent determinations. One-way ANOVA by Newman–Keuls *post hoc* test is used. ^∗∗∗^*P* < 0.001 compared with control medium. The analysis of *P* (AVOVA) indicates the following: for 30-min group, *P* < 0.001; for 120-min group, *P* < 0.001.

It has been well established that efflux of intracellular K^+^ is commonly associated with the activation of NLRP3 inflammasome in response to diverse stimuli (Muñoz-Planillo et al., 2013; Gaidt and Hornung, 2018). Furthermore, the binding of P2X_7_ purinergic receptor with extracellular ATP results in an efflux of intracellular K^+^ that induces assembly and activation of NLRP3 inflammasome in LPS-primed macrophages (Yaron et al., 2015). In the present study, LPS by itself did not modify the intracellular K^+^ concentration. However, the addition of 5 mM ATP into LPS-primed macrophages induced a significant decrease of intracellular K^+^ level, indicating the K^+^ efflux in inflammasome activation. Moreover, PA-F4 almost completely abolished the K^+^ efflux reaction (Figure 6).

### PA-F4 Constituents Inhibit LPS-Induced IL-6 Release and ATP-Induced Increase of Caspase-1 Levels in LPS-Primed Macrophages

**FIGURE 7:**
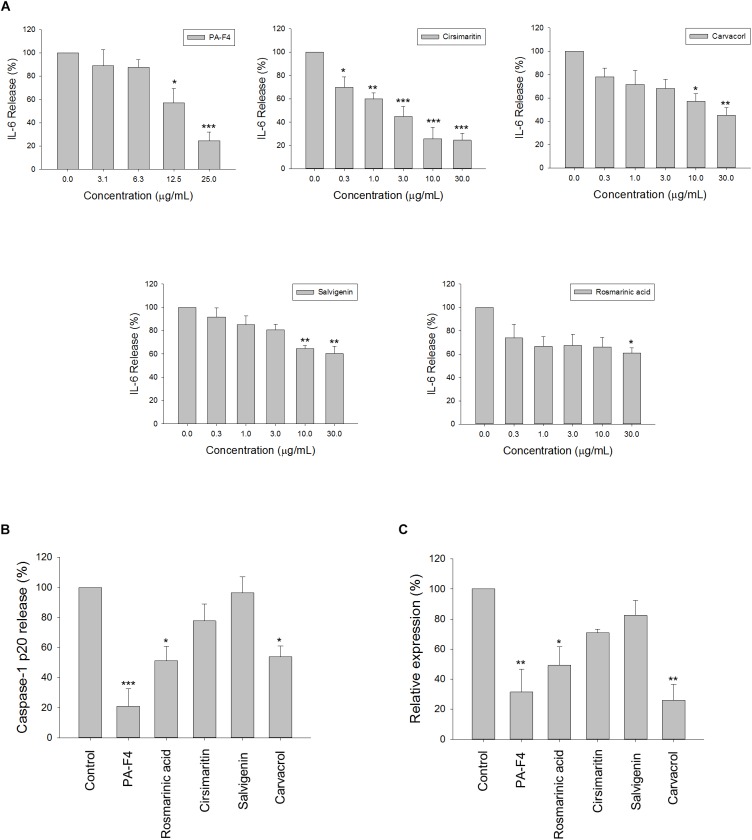
Effect of PA-F4 constituents on release reaction of IL-6 and caspase-1 and protein expression of caspase-1. THP-1 cells were seeded and differentiated by 50 nM PMA. After 48 h, cells were stimulated with 1 μg/mL LPS for 3 h for the examination of IL-6 release reaction **(A)**, or the cells were treated with 5 mM ATP for another 2 h and then, the levels of p20 caspase-1 subunit in the medium were quantified **(B)** or the p20 caspase-1 subunit expression was detected **(C)**. PAF4 (25 μg/ml), Rosmarinic acid (30 μg/ml), cirsimaritin (30 μg/ml), salvigenin (10 μg/ml), and carvacrol (30 μg/ml) were used **(B,C)**. Data are expressed as mean ± SEM of three to four independent determinations. One-way ANOVA by Newman–Keuls *post hoc* test is used. ^∗^*P* < 0.05, ^∗∗^*P* < 0.01, ^∗∗∗^*P* < 0.001 compared with control medium. The analysis of *P* (ANOVA) indicates the following: For **(A)**, PAF4, *P* < 0.001; for Cirsimaritin, *P* < 0.001; for Carvacrol, *P* = 0.005; for Salvigenin, *P* = 0.002; for Rosmarinic acid, *P* = 0.048; for **(B)**, *P* < 0.001; for **(C)**, *P* = 0.001.

There are several active constituents in PA-F4, including rosmarinic acid, cirsimaritin, salvigenin and carvacrol (Supplementary Figure S2). The effects of these constituents on LPS-induced IL-6 release and ATP-mediated caspase-1 release from LPS-primed cells were studied. The data in Figure 7A demonstrated that all of these constituents were effective in blocking IL-6 release with IC_50_ values of >30, 3.0, >30, and 22.3 μg/ml, respectively. Furthermore, cirsimaritin and carvacrol were effective on inhibiting ATP-induced release and protein expression of caspase-1 from LPS-primed cells (Figure 7B,C). The data suggested that all four constituents could, at least partly, explain PA-F4-mediated inhibitory activity on blocking the activation of NLRP3 inflammasome.

## Discussion

Natural products are one of the major resources for the research of anti-inflammatory treatment. However, many of them lack the mechanistic explanation of the action, in particular in the regulation of NLRP3 inflammasome activity which is an intracellular multimeric protein complex and plays a crucial role in the pathogenesis of various inflammatory diseases. NLRP3 inflammasome is responsible for caspase-1-dependent IL-1β processing and activation in response to a wide variety of pathogens (Cullen et al., 2015; Hayward et al., 2018). IL-1β is a pro-inflammatory cytokine implicated in inflammation, pain and autoimmune conditions (Ren and Torres, 2009). Therefore, a lot of studies suggest that blockade of NLRP3 inflammasome activation and IL-1β production is considered as a potential therapeutic strategy. A two-step activation model appears in the activation of NLRP3 inflammasome and IL-1β maturation. LPS, a major component of the outer membrane of the Gram-negative bacteria, may play a crucial role in the first step of the TLR signaling that induces NF-κB-dependent gene expression encoding NLRP3, pro-IL-1β and pro-IL-18. The second signal is the stimulation of inflammasome assembly, leading to caspase-1 activation and processing of pro-IL-1β and pro-IL-18 into active forms. Various agents including ATP, nigericin and streptolysin O are able to stimulate the step II activation (Mariathasan et al., 2006; Ren and Torres, 2009). In the step one system, IκBα is an inhibitor of the NF-κB transcription factor through blocking the nuclear localization signals (NLS) of NF-κB and its DNA binding that keeping this protein sequestered in the cytoplasm in an inactive form. In response to LPS stimulation, IκBα phosphorylation and subsequent degradation lead to unmasking of the NLS, allowing the nuclear translocation and activation of NF-κB (Piette et al., 1997). Our data showed that PA-F4 effectively inhibited LPS-induced IκBα phosphorylation and nuclear translocation of p65 NF-κB in differentiated THP-1 cells, suggesting that PA-F4 displayed an inhibitory activity on the LPS priming step.

The MAPKs including ERK, JNK and p38 MAPK are important signal transducers regulating inflammation. ERK has been suggested to play both pro- and anti-inflammatory roles depending on the inducers and cell types (Maeng et al., 2006; Ghonime et al., 2014). Notably, Ghonime et al. (2014) have reported that ERK1 (p44 MAPK) phosphorylation is critical to LPS priming process in human monocyte models. They have identified that ERK inhibition and small interfering RNA-mediated ERK1 knockdown significantly decreased the priming effect, suggesting the pro-inflammatory role of ERK, in particular ERK1. JNK serving as a pro-inflammatory MAPK has been extensively studied. Furthermore, JNK has been suggested to be a potential target for the treatment of inflammation-related diseases (Han et al., 2016). Our study totally agreed with these reports and demonstrated that the inhibition of ERK, especially ERK1, and JNK could at least partly contribute to PA-F4-mediated anti-inflammatory effect. As for p38 MAPK, it is widely involved in intracellular signaling cascades causing inflammatory responses. Accordingly, the inhibition of p38 MAPK may provide a potential strategy for treatment of inflammatory diseases (Branger et al., 2002). However, not only pro-inflammatory cytokines and chemokines, p38 MAPK also has been indicated to promote the expression of anti-inflammatory cytokines, such as IL-10, and several anti-inflammatory genes (Hammaker et al., 2014). Our data showed that PA-F4 could promote LPS-primed p38 MAPK activity although not at a significant level. However, whether the increased p38 MAPK-dependent impact preferred pro- or anti-inflammatory effects needs further elucidation.

Inflammasomes produce host defense through inducing caspase-1 activation for cytokine maturation as well as cell death. Among inflammasome family, the NLRP3 inflammasome is the most investigated given its involvement in a lot of human diseases. Most NLRs have a complex structure containing a central nucleotide-binding-and-oligomerization domain which regulates self-oligomerization, caspase activation and recruitment domain (CARD), a pyrin domain (PYD), an acidic transactivating domain or a baculovirus inhibitor repeat and carboxy-terminal leucine rich repeats possibly involved in recognition of stimuli (He et al., 2016). ASC is an adaptor protein connecting NLRP3 and pro-caspase-1 to form the inflammasome complex. PYD is responsible for the interactions between NLRP3 and ASC, and CARD is for that between ASC and pro-caspase-1 (Lu and Wu, 2015). Furthermore, both ASC dimerization and oligomerization are thought to be direct evidence for inflammasome activation (Lugrin and Martinon, 2017). This study showed that after LPS priming, the ATP application induced a dramatic increase of both ASC dimerization and oligomerization, which were inhibited by PA-F4 in a concentration-dependent manner. The data indicated that PA-F4 inhibited the step two activation of NLRP3 inflammasome. Further identification of the interaction between NLRP3 and ASC using co-immunoprecipitation showed that ATP application induced an increase of this interaction in LPS-primed cells. Furthermore, this interaction effect was completely inhibited by PA-F4, further suggesting the inhibitory activity of PA-F4 on the association mechanism and NLRP3 inflammasome activation.

The P2X_7_ receptor is a ligand-operated cation channel which opens in response to ATP binding, leading to cell depolarization and the regulation of a range of cellular processes (Khakh, 2001). It has been suggested that ATP binding to P2X_7_ receptors in inflammatory cells can lead to the open of rapid potassium-selective channels. Moreover, activated P2X_7_ receptors can trigger step two of NLRP3 inflammasome assembly and activation (Albalawi et al., 2017). Not only ATP, many agents including streptolysin O, Nigericin and maitotoxin are able to induce K^+^ efflux from the cell. The application of extracellular high K^+^ can inhibit the activation of NLRP3 inflammasome and IL-1β release (Muñoz-Planillo et al., 2013; Albalawi et al., 2017), indicating the mechanism of intracellular K^+^ efflux in the activation of NLRP3 inflammasome. Similarly, our data also showed that ATP application in LPS-primed cells induced a significant K^+^ efflux from the cells. This effect was completely inhibited by PA-F4, further validating the inhibitory effect of PA-F4 on the activation of NLRP3 inflammasome.

Rosmarinic acid, cirsimaritin, salvigenin, and carvacrol are four constituents of PA-F4. The anti-inflammatory effects of rosmarinic acid, cirsimaritin, and carvacrol but not salvigenin have been studied through the inhibition of NF-κB activation (Shin et al., 2017; Wei et al., 2018; Xiao et al., 2018). Among them, only rosmarinic acid has been reported to suppress the activation of NLRP3 inflammasome (Wei et al., 2018). Our study showed that all four PA-F4 constituents inhibited the release reaction of IL-6 indicating their anti-inflammatory activities. Furthermore, rosmarinic acid and carvacrol significantly suppressed the release reaction and protein levels of activated caspase-1 suggesting their anti-inflammasome activities.

## Conclusion

**FIGURE 8:**
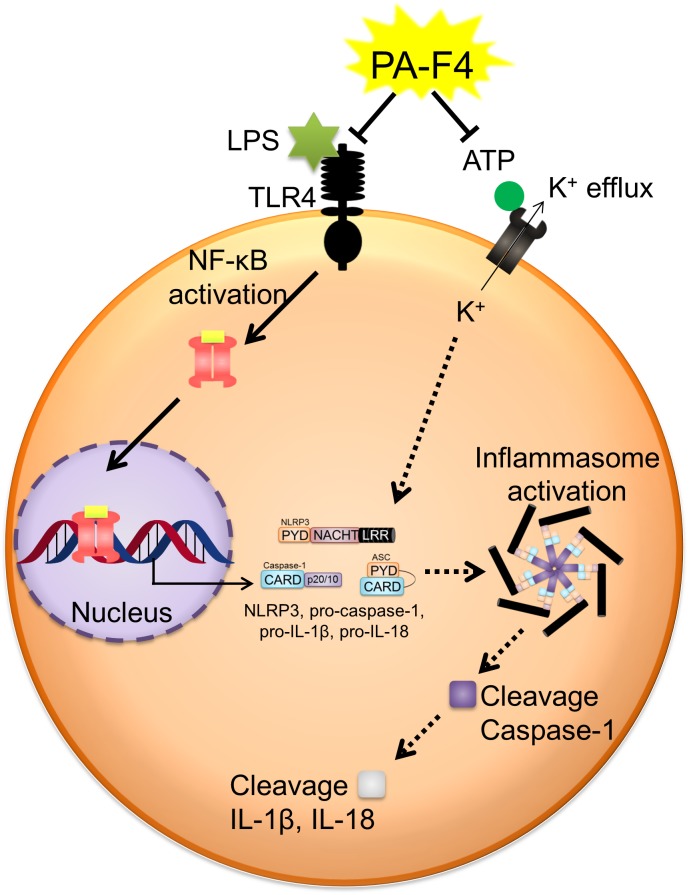
Schematic figure for PA-F4-mediated inhibitory effect on the activation of NLRP3 inflammasome. PA-F4 inhibited LPS priming step through the block of p65 NF-κB activation. It also inhibited ATP-induced signaling pathways in LPS-primed cells including the inhibition of the interaction between NLRP3 and ASC, K^+^ efflux reaction, and the release reaction of caspase-1, IL-1β, and IL-18. Taken together, the data suggest that PA-F4 shows an inhibitory activity on blocking the activation of NLRP3 inflammasome.

PA-F4 displayed an inhibitory activity on the activation of NLRP3 inflammasome (Figure 8). PA-F4 inhibited LPS priming step through the block of p65 NF-κB activation. It also inhibited ATP-induced signaling pathways in LPS-primed cells including the inhibition of both ASC dimerization and oligomerization, K^+^ efflux reaction, and the release reaction of caspase-1, IL-1β, and IL-18. Taken together, the data suggest that PA-F4 shows an inhibitory activity on blocking the activation of NLRP3 inflammasome. Furthermore, the constituents rosmarinic acid, cirsimaritin, salvigenin, and carvacrol may, at least partly, explain the inhibitory effects of inflammasome activation and inflammation.

## Author Contributions

J-HG and J-CC conceived the project and designed research. W-JL conducted experiments and analyzed data. J-HG wrote the manuscript. All authors read and approved the manuscript.

## Conflict of Interest Statement

J-HG received one research grant from Oneness Biotech Co., Ltd. (ON101NDD01). The remaining authors declare that the research was conducted in the absence of any commercial or financial relationships that could be construed as a potential conflict of interest.
