# Supplementary figures and images for: Extract From Plectranthus amboinicus Inhibit Maturation and Release of Interleukin 1β Through Inhibition of NF-κB Nuclear Translocation and NLRP3 Inflammasome Activation
Source: Front Pharmacol. 2019 May 28;10:573. doi: 10.3389/fphar.2019.00573 (PMC6546882; doi:10.3389/fphar.2019.00573)

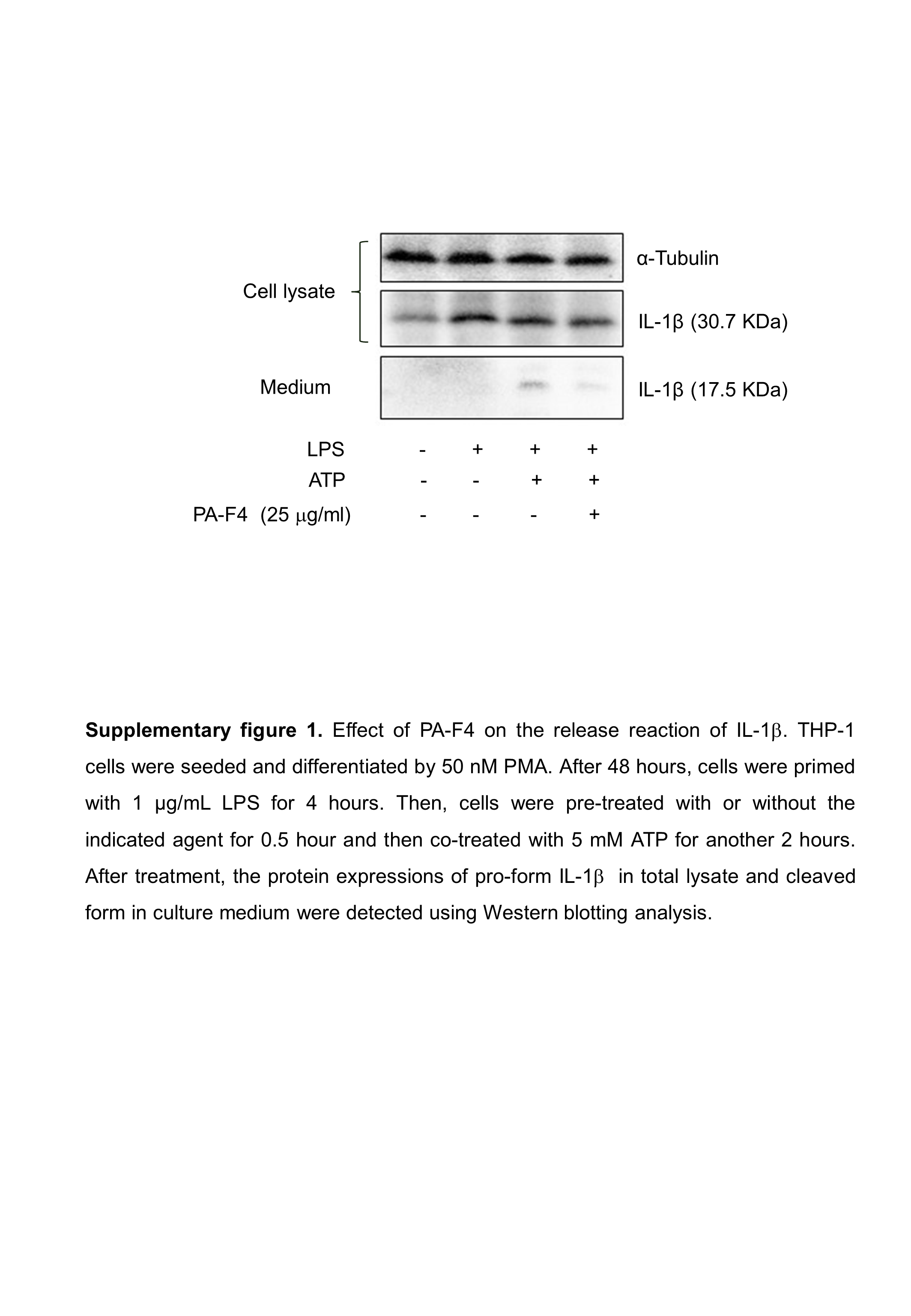

Supplement: Supplementary file 1 [file Image_1.TIF]

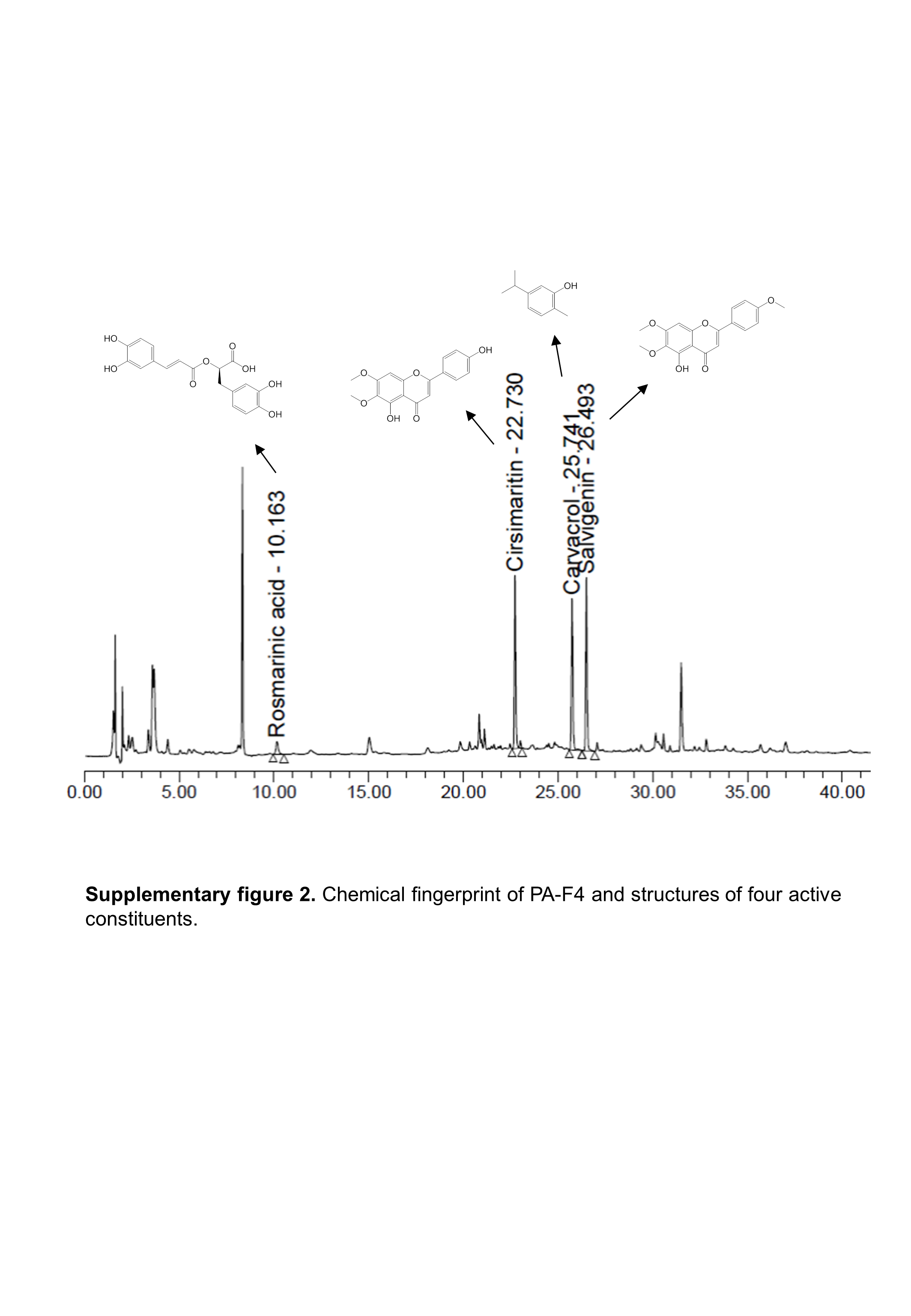

Supplement: Supplementary file 2 [file Image_2.TIF]
